# High shear stress suppresses proliferation and migration but promotes apoptosis of endothelial cells co-cultured with vascular smooth muscle cells via down-regulating MAPK pathway

**DOI:** 10.1186/s13019-019-1025-5

**Published:** 2019-12-12

**Authors:** Qiang Ji, Yu Lin Wang, Li Min Xia, Ye Yang, Chun Sheng Wang, Yun Qing Mei

**Affiliations:** 1 Department of Cardiovascular Surgery of Zhongshan Hospital, Fudan University, 180 Fenglin Road, Shanghai, 200032 China; 2 Shanghai Institute of Cardiovascular Diseases, 1609 Xietu Rd, Shanghai, 200032 China; 3 Department of Cardiothoracic surgery, Tongji Hospital of Tongji University, 389 Xincun Road, Shanghai, 200065 China

**Keywords:** Shear stress, Endothelial cells, Co-culture system, Cell proliferation, Cell apoptosis, Cell migration, Mitogen-activated protein kinase pathway

## Abstract

**Background:**

Early neointimal hyperplasia of vein graft may be ameliorated via enhancing intravenous surface shear stress. Cellular processes including proliferation, apoptosis and migration of endothelial cells (ECs) and vascular smooth muscle cells (VSMCs) may play very important roles in the process of neointimal hyperplasia of vein graft; and mitogen-activated protein kinase (MAPK) pathways including extracellular signal-regulated kinase (ERK1/2) and p38 pathways play vital roles in regulating a large variety of cellular processes. This study evaluated the impacts of shear stress and MAPK pathways on cellular processes of ECs in a co-culture system with VSMCs, and aimed to test the hypothesis that high shear stress suppresses proliferation and migration but promotes apoptosis of ECs co-cultured with VSMCs via down-regulating MAPK pathway.

**Methods:**

Primary ECs and VSMCs derived from porcine great saphenous vein were collected, respectively. 4–7 generation of cells were used as work cells. ECs and VSMCs were co-cultured and synchronized under high and low shear stress using *Parallel-Plate Flow Chamber* system. And then, ECs co-cultured with VSMCs were incubated with U0126 (ERK1/2 inhibitor) or PD98059 (p38 inhibitor) under different shear stress. Proliferation, apoptosis and migration of ECs in a co-culture system with VSMCs were detected by 4,5-dimethyl-2-thiazolyl (MTT) assay and bromodeoxyuridine (BrdU) assay, fluorescent-activated cell sorting (FACS) technique, and Transwell assay separately. Each test repeated 3 times. Additionally, protein expressions of ERK1/2 and p38 MAPK were detected by using Western blot, respectively.

**Results:**

Under higher level of shear stress condition, proliferation and migration of ECs co-cultured with VSMCs were suppressed, while cell apoptosis was promoted. And blocking ERK1/2 pathway by U0126 or blocking p38 pathway by PD98059, proliferation and migration of ECs co-cultured with VSMCs were further suppressed, while cell apoptosis was further promoted. Additionally, protein expressions of phosphorylation of ERK1/2 and p38MAPK were decreased under higher level of shear stress condition, and were further reduced by blocking ERK1/2 or p38 pathway under shear stress condition.

**Conclusions:**

High shear stress may suppress proliferation and apoptosis of ECs in a co-culture system with VSMCs but promote cell migration via down-regulating ERK1/2 and p38 MAPK pathways.

## Introduction

Coronary artery bypass grafting (CABG) is one of the most effective treatments for coronary artery disease [1]. The autologous saphenous vein is still the most frequently used bypass conduit. However, increasing studies have demonstrated that autologous saphenous vein graft is prone to severe restenosis or even occlusion (also known as vein graft failure) after bypass grafting to coronary artery system [2, 3]. Vein graft restenosis is the main cause of the failure of CABG. External vascular stent, a promising approach to prevent wall thickening and neointimal hyperplasia of vein graft and to prevent vein graft failure, is still at the experimental research stage.

Based on external vascular stent, we have developed a double-layer autologous vein graft consisting of an inner layer of autologous great saphenous vein as the vein graft and an outer layer of autologous great saphenous vein as the external reinforcement. Our previous study demonstrated that double-layer vein grafting was effective in restraining early excessive distension of vein graft and ameliorating early neointimal hyperplasia via enhancing intravenous surface shear stress through animal experiments [4]. However, the underlying molecular mechanisms of high shear stress induced by double-layer vein grafting alleviating neointimal hyperplasia of vein graft remained unknown.

Cellular processes including proliferation, apoptosis and migration may play very important roles in the process of neointimal hyperplasia of vein graft after bypass grafting to coronary artery system [5–7]. The majority of previous studies focused on evaluating the impacts of shear stress on the structure and function of endothelial cells (ECs) or vascular smooth muscle cells (VSMCs). However, few studies have been focused on evaluating the impacts of shear stress on cellular processes of ECs in a co-culture system with VSMCs.

The members of the mitogen-activated protein kinase (MAPK) family, including extracellular signal-regulated kinase (ERK1/2) and p38 MAPK, have been proposed as important signaling components mediating extra-cellar stimulation, such as physical stress, oxidative stress and mechanical stress [8, 9]. MAPK pathways play vital roles in regulating a large variety of cellular processes including proliferation, apoptosis and migration [10].

In the present study, ECs and VSMCs were co-cultured and synchronized under shear stress using *Parallel-Plate Flow Chamber* system, and then were incubated with U0126 (ERK1/2 inhibitor) or PD98059 (p38 inhibitor). Cellular processes including proliferation, apoptosis and migration of ECs co-cultured with VSMCs were detected, respectively; and protein expressions of ERK1/2 and p38 MAPK were determined, respectively. This study evaluated the impacts of shear stress and MAPK pathways on proliferation, apoptosis and migration of ECs co-cultured with VSMCs, and aimed to test the hypothesis that high shear stress suppresses proliferation and migration but promotes apoptosis of ECs co-cultured with VSMCs via down-regulating MAPK pathway.

## Materials and methods

### Study protocol

This study protocol was approved by the ethics committee of Tongji Hospital of Tongji University (No. LL(H)-0–14-11) and was consistent with the *Declaration of Helsinki*.

According to our published results, double-layer vein grafting compared with conventional single-layer vein grafting may have contributed towards a rise in intravenous surface shear stress of vein graft. The details of double-layer autologous saphenous vein grafting in a porcine model and calculation of intravenous surface shear stress of vein graft were shown in the Additional file 1. The amount of shear stress detected in our published animal experiment with double-layer vein grafting was set as SS1 (higher shear stress) and the amount of shear stress detected in single-layer vein grafting was set as SS2 (lower shear stress) [4, 11].

To evaluate the impacts of shear stress on cellular processes, ECs and VSMCs derived from porcine great saphenous vein were collected, respectively, and then were co-cultured and synchronized under different levels of shear stress condition using *Parallel-Plate Flow Chamber* system with shear stress gradients. To evaluate the impacts of MAPK pathway on cellular processes, ECs co-cultured with VSMCs under shear stress condition were incubated with U0126 (ERK1/2 inhibitor) or PD98059 (p38 inhibitor).

The experimental groups settings were as follows: Group SS1 (cells were subjected to high shear stress of SS1), Group SS2 (cells were subjected to low shear stress of SS2), Group SS1 + U0126 (cells were subjected to high shear stress of SS1 and incubated with U0126), Group SS2 + U0126 (cells were subjected to low shear stress of SS2 and incubated with U0126), Group SS1 + PD98059 (cells were subjected to high shear stress of SS1 and incubated with PD98059), and Group SS2 + PD98059 (cells were subjected to low shear stress of SS2 and incubated with PD98059). Co-cultured cells without shear stress condition or MAPK inhibitors were used as the blank control (Group Con).

Proliferation, apoptosis and migration of ECs co-cultured with VSMCs in all groups were detected by using 4,5-dimethyl-2-thiazolyl (MTT) assay and bromodeoxyuridine (BrdU) assay, fluorescent-activated cell sorting (FACS) technique, and Transwell assay separately. Protein expressions of ERK1/2 and p38 MAPK were determined by using Western blot (WB), respectively. Each test repeated 3 times.

### Isolation and identification of ECs and VSMCs

Studies were performed with five healthy Shanghai white pigs (weight 20–25 kg), which were provided by Shanghai Multi-Bio-Sci-Tech Co. Ltd. (license: SCXK2005–0002). Anesthesia was performed with intravenous ethaminal sodium (30 mg/kg). Fresh porcine great saphenous veins were digested with a mixture of 0.1% collagenase and 0.125% trypsin (Sigma company) (1:4), and the endothelial cell suspension was cultured in M199 medium containing 20% fetal bovine serum (Gibco) and acidic fibroblast growth factor (aFGF, Sigma). Cells of the 2nd generation were stained with vWF antibody, a specific marker of ECs, to determine the percentage of ECs. And 2-(4-Amidinophenyl)-6-indolecarbamidine dihydrochloride (DAPI) was used to indicate nucleus.

VSMCs were collected through adopting explant culture and were cultured in the DMEM medium containing 10% fetal bovine serum, and the solution was changed once every 2 to 3 days. Cells of the 2nd generation were stained with anti-α-actin antibody to identify VSMCs. And DAPI was used to indicate nucleus. Pictures were taken under the fluorescence microscope (Olympus, Japan).

### Co-culture of ECs and VSMCs and the flow chamber system

**Fig. 1:**
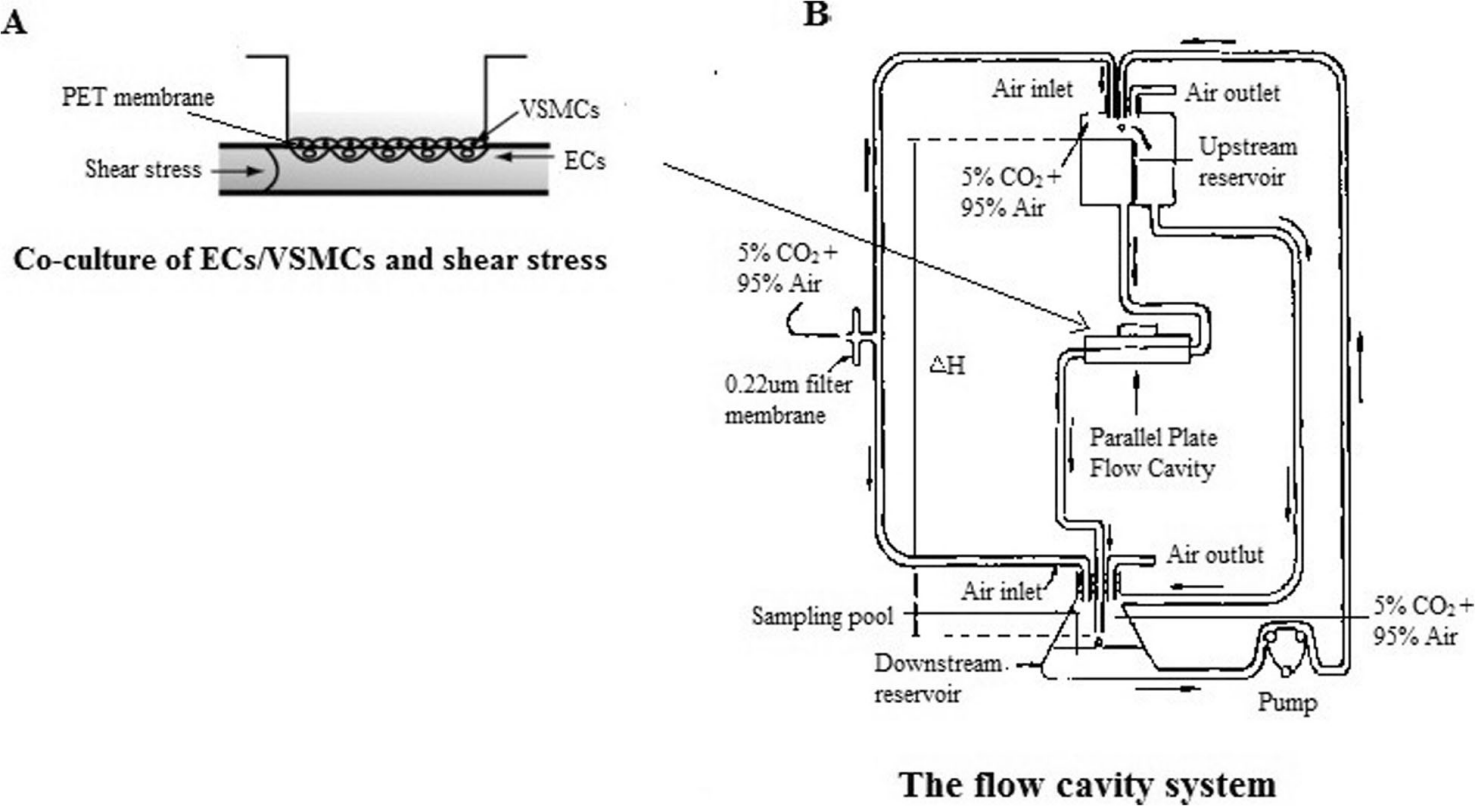
Co-culture of ECs and VSMCs and the flow chamber system. **a**. Co-culture of ECs and VSMCs under shear stress condition. **b**. The flow chamber system was mainly composed of three parts: parallel-plate flow cavity for co-culture of ECs/VSMCs, liquid perfusion system, and temperature and acidity/alkalinity control device

Four to seven generation of cells were used as work cells. ECs (the concentration of 2*10^5^ cells/ml) were planted on the outside of PET membrane (Falcon Co., Ltd.) within the co-culture cup. The membrane was reversed after ECs adhered completely (about 3 to 4 h), and VSMCs were planted on the inner surface of the membrane with the concentration of 1*10^5^ cells/ml. And then, the PET membrane was inserted into the matching 6-well culture plate for co-culture (as shown in Fig. 1a). After VSMCs and ECs grew to 80% fusion, the cell growth was blocked in the same cycle by synchronizing using DMEM medium containing 1% fetal bovine serum and M199 medium containing 1% fetal bovine serum for 12 h. Co-cultured cells were first 1% serum starved for 12 h, and then incubated for 30 min with U0126 or PD98059, respectively, before finally being exposed to different levels of shear stress.

The flow cavity system (as shown in Fig. 1b) used in this experiment was mainly composed of three parts: parallel-plate flow cavity for co-culture of ECs and VSMCs, liquid perfusion system, and temperature and acidity/alkalinity control device. The different levels of laminar shear stress acting on the ECs surface occurred when the perfusion fluid flowed through the flow cavity. The temperature was maintained at 37 °C; and the pH was held constant by using binary-component gas (5% carbon dioxide + 95% air).

### Cell proliferation

Both MTT assay and BrdU assay were used to detect ECs proliferation. Experiments were taken under the manufacturers’ instructions. Briefly, for MTT assay, ECs were seeded in a 96-well plate at a density of 3000 cells/well. At indicated time points, MTT was added into each well at a final concentration of 5 mg/ml for 4 h. The medium was then removed and the cells were incubated for 15 min with 100 ml of acidic isopropanol (0.08 N HCl) to dissolve the formazan crystals. The absorbance of the MTT formazan was determined at 490 nm in an enzyme-linked immunosorbent assay (ELISA) reader (Bio-Rad, USA). For BrdU assay, cells from different groups were incubated in 1 M HCl for 10 min on ice to break open the DNA structure. This is follower by 2 M HCl for 10 min at room temperature, then 20 min at 37 °C. Immediately after the acid incubations, neutralize by incubating the samples in 0.1 M borate buffer for 10 min at room temperature. The cells were washed in PBS (pH = 7.4), 0.1% Triton X-100 3 times, 5 min per wash. Continue with standard staining procedure as described in the immunohistochemistry protocol.

### Cell apoptosis

FACS technique was applied to determine whether endothelial cell apoptosis was affected by shear stress and/or MAPK inhibitors. Cells were trypsinized and washed twice with cold PBS (the same as above). And then, cells were collected, washed and stained with Annexin-V (Invitrogen, US) for 30 min at room temperature. Cells were then washed. PI was added 5 min before subjected to FACS analysis.

### Cell migration

Transwell assay was used to determine whether endothelial cell migration was affected by shear stress and/or MAPK inhibitors. This assay involved a two-compartment system where cells were induced to migrate from an upper compartment through a porous membrane into a lower compartment following the gradient of a chemokine. To determine the extent to which cells have migrated, the filter inserts are removed from the culture wells, the cells that have not migrated are physically cleared from the top surface of the filter, and the remaining cells are fixed and stained. Each Transwell chamber was observed three visual fields randomly and endothelial cells were counted.

### Protein expression

Protein expression was analyzed by using western blot. Total cellular protein was extracted in radio immunoprecipitation assay (RIPA) buffer (containing 1% Triton X-100, 1% deoxycholate, and 0.1% SDS). Protein was quantified using the BCA (Pierce biotechnology, Rockford, US) according to the manufacturer’s instructions. Equal amounts of protein were separated through SDS-PAGE and transferred to a PVDF membrane. Membrane were blocked in 5% milk and then incubated with antibodies against of phospho-ERK1/2 (1:1000 dilution; CST, US), phospho-p38 (1:1000 dilution; CST, US), total ERK1/2 (1:1000 dilution; CST, US), total p38 (1:1000 dilution; CST, US) and GAPDH (1:1000 dilution; Santa Cruze, US) over night. Membranes were washed and incubated with secondary antibody conjugated to HRP (Santa Cruze, US). Immunoreactive bands were visualized by chemiluminescence. Results were analyzed by densitometry.

### Statistical analysis

Continuous variables were expressed as mean ± standard deviation and were compared between different groups using one-way analysis of variance and Dunnett post hoc test. All statistical tests were two-sided. Results were considered statistically significant at a level of *p* less than 0.05. All analyses were performed with the SPSS statistical package version 20.0 (SPSS Inc., Chicago, IL, USA).

## Results

### Isolation and identification of ECs and VSMCs

**Fig. 2:**
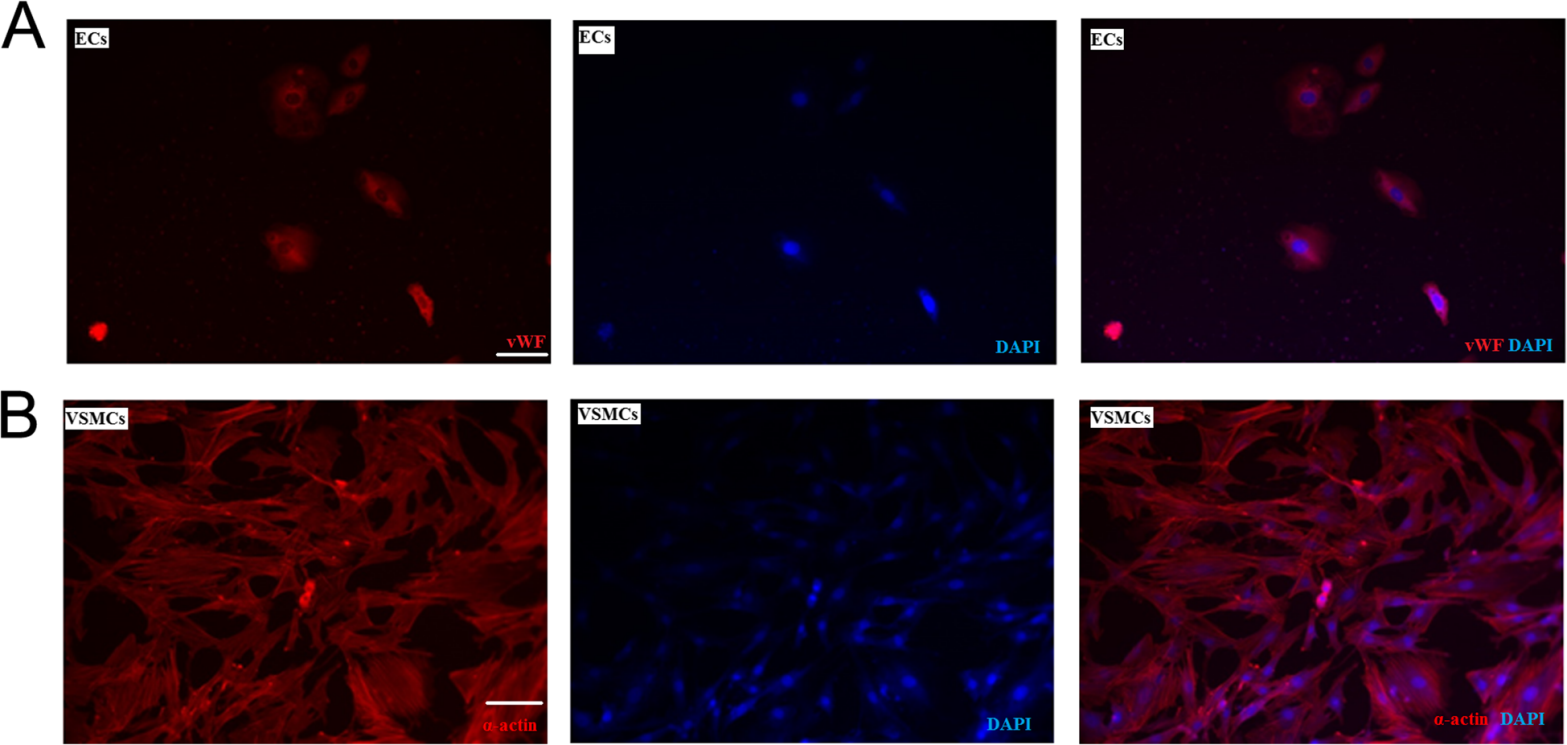
Isolation and identification of ECs and VSMCs. **a**. vWF assay was used to identify ECs (bar = 50 μm). **b**. α-actin assay was used to identify VSMCs (bar = 50 μm)

The result of immunofluorescence showed the positive cells of vWF were found in over 95% of the ECs of the second generation of culture, and fluorescence staining could be seen in the cytoplasm (as shown in Fig. 2a), indicating that ECs were collected successfully. As shown in Fig. 2b, the positive cells of α-actin were found in more than 90% of the VSMCs of second generation of culture, and a large number of myofilament could be seen in the cytoplasm, suggesting that the VSMCs from porcine great saphenous vein were collected successfully.

### ECs proliferation

**Fig. 3:**
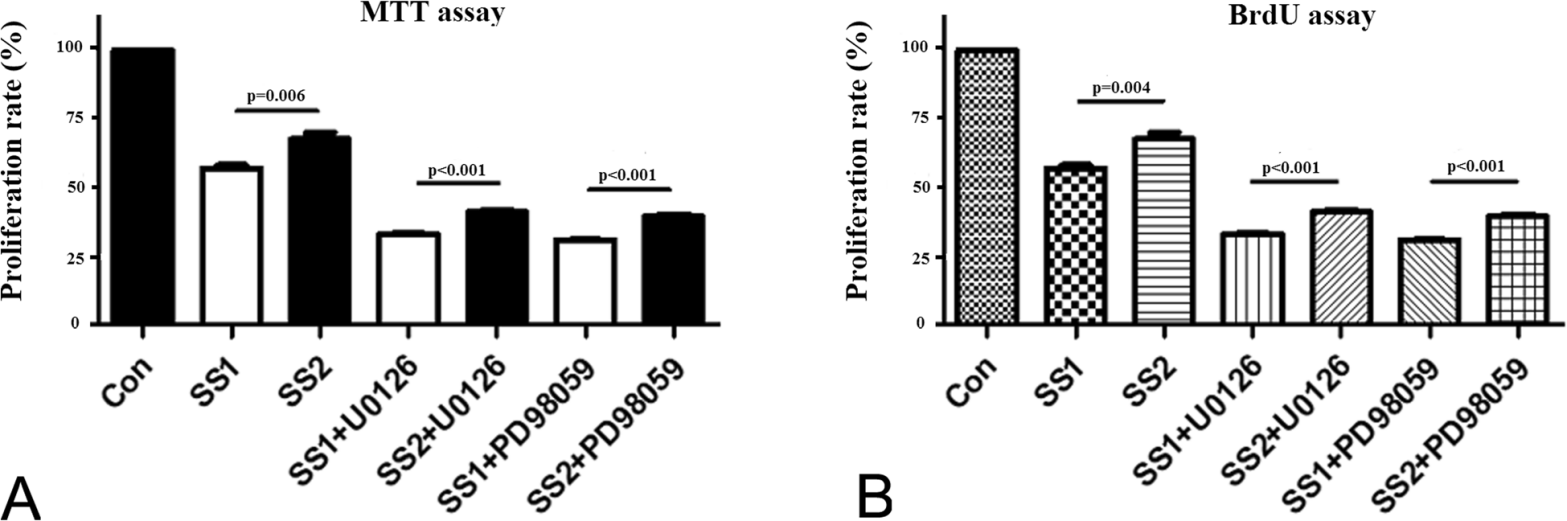
Detection of ECs proliferation. **a**. MTT assay was used to detect ECs proliferation. Significant differences were observed among the group Con, the group SS1, and the group SS1 + U0126 (*p* < 0.001). And, significant differences were observed among the group Con, the group SS1, and the group SS1+ PD98059 (*p* < 0.001). **b**. BrdU assay was used to detect ECs proliferation. Significant differences were observed among the group Con, the group SS1, and the group SS1 + U0126 (*p* < 0.001). And, significant differences were observed among the group Con, the group SS1, and the group SS1+ PD98059 (*p* < 0.001)

As shown in Fig. 3a, results of MTT assay showed that significant differences were found with regard to proliferation rate of ECs between the group SS1 and the group SS2, between the group SS1 + U0126 and the group SS2 + U0126, and between the group SS1 + PD98059 and the group SS2 + PD98059 (*p* = 0.006, *p* < 0.001, and *p* < 0.001, respectively), suggesting higher shear stress compared with lower shear stress may suppress ECs proliferation. Significant differences were observed with regard to proliferation rate of ECs among the group Con, the group SS1, and the group SS1 + U0126 (*p* < 0.001), indicating higher shear stress may suppress ECs proliferation and blocking ERK1/2 pathway may further suppress proliferation of ECs co-cultured with VSMCs under shear stress condition. There were significant differences were observed with regard to proliferation rate of ECs among the group Con, the group SS1, and the group SS1+ PD98059 (*p* < 0.001), suggesting blocking p38MAPK pathway may also further suppress proliferation of ECs co-cultured with VSMCs under shear stress condition. The similar results were observed in BrdU assay (as shown in Fig. 3b).

### ECs apoptosis

**Fig. 4:**
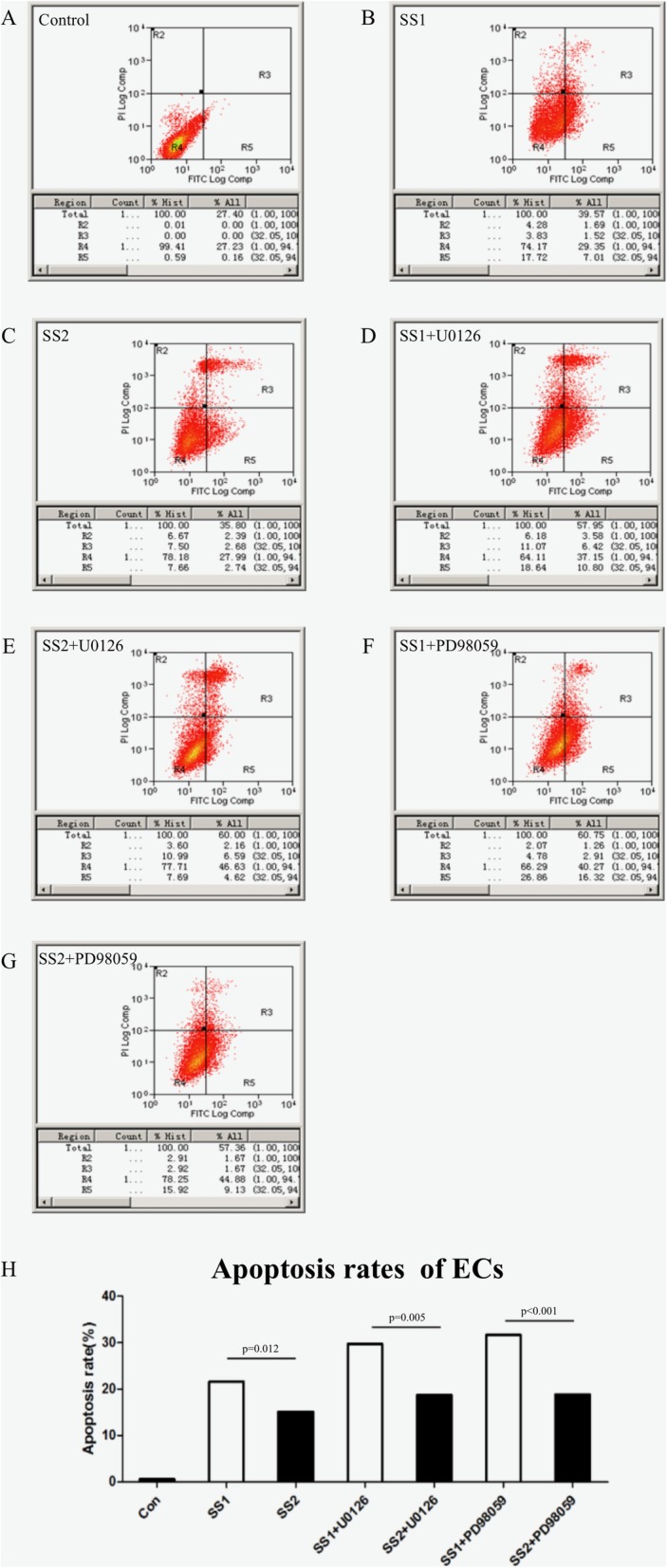
Detection of ECs apoptosis. **a-g**. Apoptosis was quantified through FACS analysis after staining with Annexin V and PI. The Annexin V+/PI- cells appeared early in the apoptotic process. The viable cells were Annexin V−/PI-. **h**. Apoptosis rate of ECs in all the 7 groups. Significant differences were observed among the group Con, the group SS1, and the group SS1 + U0126 (*p* < 0.001). And, significant differences were observed among the group Con, the group SS1, and the group SS1+ PD98059 (*p* < 0.001)

As shown in Fig. 4, significant differences were found in apoptosis rates between the group SS1 and the group SS2, between the group SS1 + U0126 and the group SS2 + U0126, and between the group SS1 + PD98059 and the group SS2 + PD98059 (*p* = 0.012, *p* = 0.005, and *p* < 0.001, respectively), indicating higher shear stress compared with lower shear stress may contribute to promoting apoptosis of ECs in a co-culture system with VSMCs. Significant differences were observed in apoptosis rates among the group Con, the group SS1, and the group SS1 + U0126 (*p* < 0.001), suggesting higher shear stress may promote ECs apoptosis and blocking ERK1/2 pathway may further promote ECs co-cultured with VSMCs under shear stress condition. There were significant differences in ECs apoptosis rates among the group Con, the group SS1, and the group SS1+ PD98059 (*p* < 0.001), suggesting blocking p38MAPK pathway may further promote apoptosis of ECs co-cultured with VSMCs under shear stress condition.

### ECs migration

**Fig. 5:**
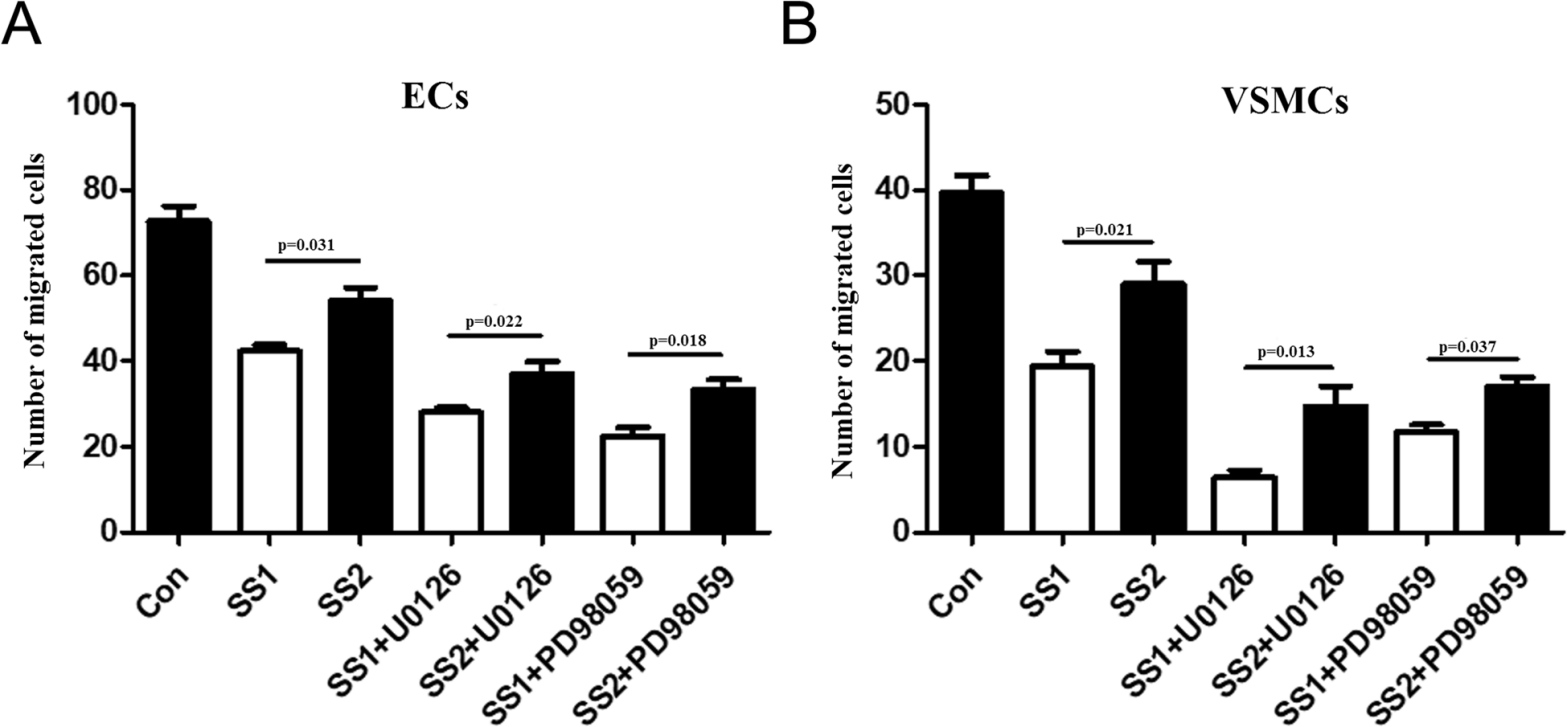
Detection of ECs migration. **a**. Transwell assay was used to determine migration of ECs. Significant differences were observed among the group Con, the group SS1, and the group SS1 + U0126 (*p* = 0.005). And, significant differences were observed among the group Con, the group SS1, and the group SS1+ PD98059 (*p* < 0.001). **b**. Transwell assay was used to determine migration of VSMCs

As shown in Fig. 5, significant differences were found in term of the number of migrated ECs between the group SS1 and the group SS2, between the group SS1 + U0126 and the group SS2 + U0126, and between the group SS1 + PD98059 and the group SS2 + PD98059 (*p* = 0.031, *p* = 0.022, and *p* = 0.018, respectively), suggesting higher shear stress compared with lower shear stress may suppress the migration of ECs in a co-culture system with VSMCs. Significant differences were observed in term of the number of migrated ECs among the group Con, the group SS1, and the group SS1 + U0126 (*p* = 0.005), suggesting higher shear stress may suppress ECs migration and blocking ERK1/2 pathway may further suppress migration of ECs co-cultured with VSMCs under shear stress condition. There were significant differences in the number of migrated ECs among the group Con, the group SS1, and the group SS1+ PD98059 (*p* < 0.001), suggesting blocking p38MAPK pathway may further suppress migration of ECs co-cultured with VSMCs under shear stress condition.

### Protein expression of MAPK

**Fig. 6:**
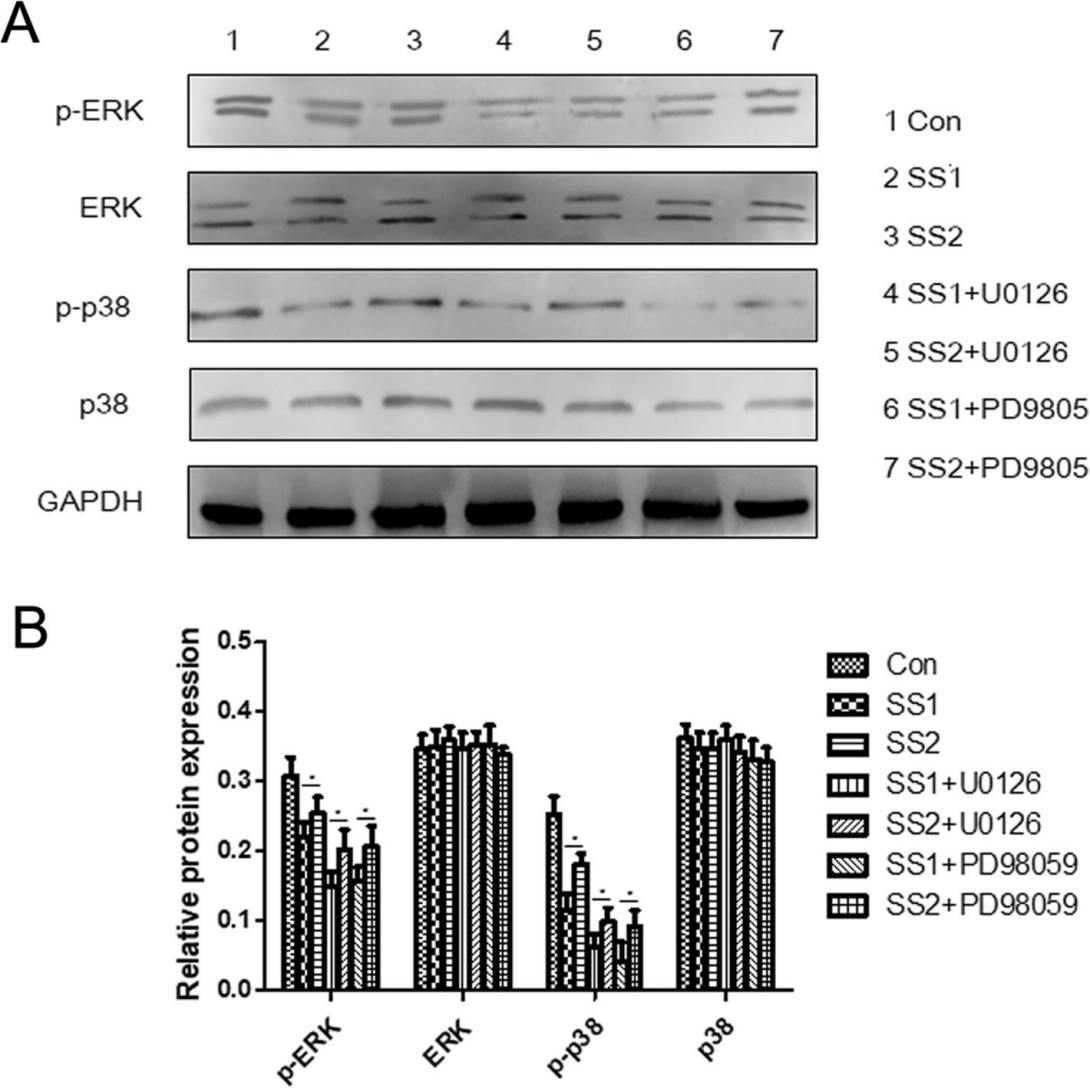
Protein expression of ERK1/2 and p38. **a** Western blot was used to determine the protein expressions of ERK1/2 and p38, and p-ERK1/2 and p-p38. **b** Quantification of Western blot results. * *p* < 0.05. Significant differences were observed in the expression of p-ERK1/2 among the group Con, the group SS1, and the group SS1+U0126 (*p* < 0.001), and significant differences were observed in the expression of p-p38 among the group Con, the group SS1, and the group SS1+ PD98059 (*p* < 0.001). No significant differences were recorded among the 7 groups with regard to the expression of ERK1/2 and p38, respectively (*p* = 0.554 and *p* = 0271, respectively)

As shown in Fig. 6, significant differences were found in the expression of phosphorylation of ERK1/2 between the group SS1 and the group SS2, between the group SS1 + U0126 and the group SS2 + U0126, and between the group SS1 + PD98059 and the group SS2 + PD98059 (*p* = 0.041, *p* = 0.028, and *p* = 0.035, respectively), suggesting higher shear stress compared with lower shear stress may decrease the expression of phosphorylation of ERK1/2. Significant differences were observed in term of the expression of phosphorylation of ERK1/2 among the group Con, the group SS1, and the group SS1 + U0126 (*p* < 0.001), suggesting higher shear stress may decrease the expression of phosphorylation of ERK1/2 and blocking ERK1/2 pathway may further decrease the expression of phosphorylation of ERK1/2 under shear stress condition. Similarly, higher shear stress compared with lower shear stress may decrease the expression of phosphorylation of p38, and blocking p38MAPK pathway may further decrease the expression of phosphorylation of p38 under shear stress condition. Additionally, no significant differences were recorded among the 7 groups with regard to the expression of ERK1/2 and p38, respectively (*p* = 0.554 and *p* = 0271, respectively), suggesting that shear stress and blocking MAPK pathway had no impacts on the expression of ERK1/2 or p38.

## Discussion

An important finding of this study is that higher shear stress compared with lower shear stress may suppress proliferation and migration but promote apoptosis of ECs in a co-culture system with VSMCs. Shear stress, the tangential hemodynamic force, has been implicated in alteration of the structure and functional properties of ECs at the cellular and molecular levels [11]. It is widely believed that wall shear stress of vein graft plays a central role in regulation of endothelial cell inflammatory responses and the pathogenesis of atherosclerosis after bypass grafting to coronary artery system. High shear stress may induce an anti-inflammatory status in endothelial cell, which is partially mediated by the production of proteins and transcription factors which can suppress different pro-inflammatory signaling pathways [12, 13]. In this study, results of both MTT and BrdU assays showed that higher shear stress compared with lower shear stress may suppress ECs proliferation, FACS analysis showed that higher shear stress compared with lower shear stress may promote ECs apoptosis, and Transwell assay showed that higher shear stress compared with lower shear stress may suppress ECs migration, all of which indicating that high shear stress may suppress proliferation and migration of ECs co-cultured VSMCs but promote cell apoptosis. This study implied that higher shear stress instead of lower shear stress was beneficial for prevention and treatment of neointimal hyperplasia of vein graft. Souilhol and colleagues [14] have reported that low shear stress promoted vascular dysfunction and atherosclerosis; conversely, high shear stress was protective. This evidence was in line with the results of this study. Additionally, Liu and colleagues [15] found that gradually increasing shear stress could improve EC retention on vascular grafts. This finding was consistent with that in this study.

Another important finding of this study is that higher shear stress suppressing proliferation and migration of ECs co-cultured with VSMCs but promote cell apoptosis may be via inactivation of ERK1/2 and p38MAPK pathways. MAPK pathway, which is activated in a variety of tissues, is activated by a range of growth factors, cytokines and cellular stresses, and is responsible for transducing extracellular signals to the cytoplasm and nucleus. A previous study has reported that shear stress may induce activation of p38 MAPK and production of IL-1β in human bone marrow-derived mesenchymal stem cells [16]. ERK1/2, JNK1/2 and p38MAPK are all involved in low shear stress-induced IL-8 gene expression in ECs [17]. Besides, lower shear stress may induce oxidative damage and cell migration via MAPK pathway [18, 19]. All those studies suggested lower shear stress was pro-inflammatory and detrimental. This study showed that higher shear stress compared with lower shear stress may suppress ECs proliferation and migration but promote cell apoptosis and decrease activation of ERK1/2 and p38; blocking ERK1/2 and p38 pathways may further suppress proliferation and migration of ECs co-cultured with VSMCs under shear stress condition but promote cell apoptosis. This study suggested that higher shear stress suppressing ECs proliferation and migration and promoting apoptosis may be via inactivation of ERK1/2 and p38 MAPK pathways. Previously, Berk and colleagues [16] have reported that high shear stress is atheroprotective, which may modulate TNF effects on EC by inhibiting TNF-mediated activation of MAP kinases. This finding was consistent with that in this study. Recently, Zakkar and colleagues [13] summarized available evidences regarding the effect of shear stress on ECs and the regulation of MAPK, and demonstrated that high shear stress may be beneficial for cellular processes of ECs via suppressing different pro-inflammatory signaling pathways. This evidence was in line with the results of this study.

Of note, a previous study [20] has reported that long-term high shear stress may increase phosphorylation of multiple MAPK species in cultured human aortic endothelial cells. Long-term impacts of high shear stress on cellular processes of ECs and its molecular mechanisms need further studies.

It is believed that hemodynamics environmental change is an initiation factor of neointimal hyperplasia of vein graft, and neointimal hyperplasia of vein graft is responsible for vein graft failure. Our previous study demonstrated that double-layer vein grafting compared with conventional single-layer vein grafting was effective in restraining early excessive distension of vein graft and ameliorating early neointimal hyperplasia via enhancing intravenous surface shear stress in animal experiments [4]. The current study found that higher shear stress compared with lower shear stress may suppress proliferation and migration of ECs in a co-culture system with VSMCs but promote cell apoptosis, and higher shear stress suppressing ECs proliferation and migration but promoting apoptosis may be via inactivation of ERK1/2 and p38 MAPK pathways. This study implies that increase of wall shear stress and inactivation of MAPK pathway may be promising strategies for preventing and treating vein graft failure. Additionally, this study may contribute to increasing the understanding of the mechanisms of vein graft failure. More importantly, this study provides experimental evidences for the clinical application of double-layer vein grafting, however, further studies are needed.

## Conclusion

This study demonstrated that high shear stress may suppress proliferation and apoptosis of ECs in a co-culture system with VSMCs but promote cell migration via down-regulating ERK1/2 and p38 MAPK pathways. This study implies that increase of wall shear stress and inactivation of MAPK pathway may be promising strategies for preventing and treating vein graft failure. This study provides experimental evidences for the clinical application of double-layer vein grafting, however, further studies are needed.

## Supplementary information

**Additional file 1.** Details of double-layer vein grafting and calculation of wall shear stress.

## Data Availability

The datasets used in the current study are available from the corresponding author or the first author on reasonable request.

## Abbreviations

BrdU: Bromodeoxyuridine
CABG: Coronary artery bypass grafting
ECs: Endothelial cells
ERK1/2: Extracellular signal-regulated kinase
FACS: Fluorescent-activated cell sorting
MAPK: Mitogen-activated protein kinase
MTT: 4,5-dimethyl-2-thiazolyl
VSMCs: Vascular smooth muscle cells
WB: Western blot
