# Supplementary material for: High shear stress suppresses proliferation and migration but promotes apoptosis of endothelial cells co-cultured with vascular smooth muscle cells via down-regulating MAPK pathway
Source: J Cardiothorac Surg. 2019 Dec 12;14:216. doi: 10.1186/s13019-019-1025-5 (PMC6909635; doi:10.1186/s13019-019-1025-5)
Supplement: Supplementary file 1 — Additional file 1. Details of double-layer vein grafting and calculation of wall shear stress. [file 13019_2019_1025_MOESM1_ESM.docx]

**Supplementary material**

1. **Double-layer vein grafting and single-layer vein grafting**

Studies were performed with healthy Shanghai white pigs (weight 20-25 kg). Anesthesia was performed with intravenous Ethaminal sodium (15 to 30mg/kg). Animals were allowed to ventilate spontaneously throughout the procedures. Heparin sodium (1mg/kg) was administered intravenously and a single dose of 250mg of benzyl penicillin was administered intramuscularly before skin incision was given.

**Double-layer vein grafting:** the great saphenous vein around the ankle, of about 4cm long (segment a), was harvested using the “no touch” technique and was employed as the ‘vein graft’ while the great saphenous vein around the homolateral knee joint, of about 6cm long (segment b), also harvested by the “no touch” technique, was employed as the ‘external vein graft support’. The vein was removed from the animal, rinsed in iso-osmotic sodium chloride solution (0.9g/L) containing 2IU/mL heparin and 50µg/mL glyceryl trinitrate and stored in the same solution at room temperature (23°C) until needed. Homolateral femoral artery of about 8 cm long was freed from surrounding tissues using the “no touch” technique. Subsequently, a 4cm segment of the femoral artery was excised between vascular clamps, beveling the cut ends obliquely to 45°. The great saphenous vein (segment b) surrounded the proximal end of the femoral artery and the great saphenous vein (segment a) was reversed and an end-to-end anastomosis of the vein to the femoral artery was carried out using a continuous 7-0 Prolene suture. Before the second anastomosis was performed, the vascular clamps were removed and the vein graft was perfused at arterial pressure. Finally, the vein graft was externally covered with the great saphenous vein (segment b) as shown in Figure 1.


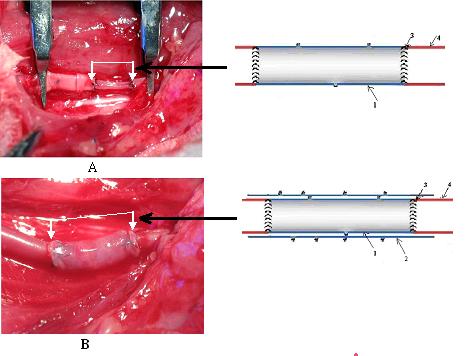


The great saphenous vein (1, vein graft) was transplanted into the femoral artery (4) though an end-to-end anastomosis (3), and then it was coated with another great saphenous vein (2, external vein graft support).

**Figure 1 Double-layer vein grafting**

**Single-layer vein grafting**: the great saphenous vein around the ankle, of about 4cm long (segment a), harvested using the “no touch” technique was employed as the ‘vein graft’. The vein was rinsed in iso-osmotic sodium chloride solution (0.9g/L) containing 2IU/mL heparin and 50µg/mL glyceryl trinitrate, and stored in the same solution at room temperature (23°C) until needed. Homolateral femoral artery of about 8cm long was freed from surrounding tissues using the “no touch” technique. Subsequently, a 4cm segment of the femoral artery was excised between vascular clamps, beveling the cut ends obliquely to 45°. Finally, the great saphenous vein was reversed and an end-to-end anastomosis of the vein to the femoral artery was carried out using a continuous 7-0 Prolene suture. Before the second anastomosis was performed, the vascular clamps were removed and the vein graft was perfused.

After surgery, all pigs were sent back to the laboratory animal center of the Tongji Hospital (clean level, license: SYXK 2007-0031). Aspirin tablet was administered every night as from the second night after the procedure.

1. **Calculation of wall shear stress**

The animals were anesthetized as already described above. The wounds were opened, blood flow volume within the middle sections of the vein grafts measured and blood flow velocity waveform detected using an electromagnetic flowmeter (NIHON KOHOEN Corporation, Japan). The diameter, blood flow volume and velocity within the vein grafts were each measured four times by an experienced operator and an average of each parameter was obtained respectively.

Wall shear stress in middle section of the vein graft was calculated according to the following formula (fluid viscosity is taken as 0.058):

Wall shear stress =

According to the formula, wall shear stress in middle section of the vein graft was inversely proportional to the inner diameter of the vein graft. Comparing with single-layer vein grafting, double-layer vein grafting restrains early excessive distension of vein graft and reduces the inner diameter of the vein graft, consequently, increases wall shear stress.
